# Heating up the roof of the world: tracing the impacts of *in-situ* warming on carbon cycle in alpine grasslands on the Tibetan Plateau

**DOI:** 10.1093/nsr/nwae371

**Published:** 2024-11-26

**Authors:** Yuxuan Bai, Yunfeng Peng, Dianye Zhang, Guibiao Yang, Leiyi Chen, Luyao Kang, Wei Zhou, Bin Wei, Yuhong Xie, Yuanhe Yang

**Affiliations:** State Key Laboratory of Vegetation and Environmental Change, Institute of Botany, Chinese Academy of Sciences, Beijing 100093, China; China National Botanical Garden, Beijing 100093, China; State Key Laboratory of Vegetation and Environmental Change, Institute of Botany, Chinese Academy of Sciences, Beijing 100093, China; China National Botanical Garden, Beijing 100093, China; State Key Laboratory of Vegetation and Environmental Change, Institute of Botany, Chinese Academy of Sciences, Beijing 100093, China; China National Botanical Garden, Beijing 100093, China; State Key Laboratory of Vegetation and Environmental Change, Institute of Botany, Chinese Academy of Sciences, Beijing 100093, China; China National Botanical Garden, Beijing 100093, China; State Key Laboratory of Vegetation and Environmental Change, Institute of Botany, Chinese Academy of Sciences, Beijing 100093, China; China National Botanical Garden, Beijing 100093, China; State Key Laboratory of Vegetation and Environmental Change, Institute of Botany, Chinese Academy of Sciences, Beijing 100093, China; China National Botanical Garden, Beijing 100093, China; College of Resources and Environment, University of Chinese Academy of Sciences, Beijing 100049, China; State Key Laboratory of Vegetation and Environmental Change, Institute of Botany, Chinese Academy of Sciences, Beijing 100093, China; China National Botanical Garden, Beijing 100093, China; College of Resources and Environment, University of Chinese Academy of Sciences, Beijing 100049, China; State Key Laboratory of Vegetation and Environmental Change, Institute of Botany, Chinese Academy of Sciences, Beijing 100093, China; China National Botanical Garden, Beijing 100093, China; College of Resources and Environment, University of Chinese Academy of Sciences, Beijing 100049, China; State Key Laboratory of Vegetation and Environmental Change, Institute of Botany, Chinese Academy of Sciences, Beijing 100093, China; China National Botanical Garden, Beijing 100093, China; College of Resources and Environment, University of Chinese Academy of Sciences, Beijing 100049, China; State Key Laboratory of Vegetation and Environmental Change, Institute of Botany, Chinese Academy of Sciences, Beijing 100093, China; China National Botanical Garden, Beijing 100093, China; College of Resources and Environment, University of Chinese Academy of Sciences, Beijing 100049, China

**Keywords:** carbon cycle, climate warming, grassland ecosystem, soil carbon dynamics, vegetation growth, warming experiment

## Abstract

Climate warming may induce substantial changes in the ecosystem carbon cycle, particularly for those climate-sensitive regions, such as alpine grasslands on the Tibetan Plateau. By synthesizing findings from *in-situ* warming experiments, this review elucidates the mechanisms underlying the impacts of experimental warming on carbon cycle dynamics within these ecosystems. Generally, alterations in vegetation structure and prolonged growing season favor strategies for enhanced ecosystem carbon sequestration under warming conditions. Whilst warming modifies soil microbial communities and their carbon-related functions, its effects on soil carbon release fall behind the increased vegetation carbon uptake. Despite the fact that no significant accumulation of soil carbon stock has been detected upon warming, notable changes in its fractions indicate potential shifts in carbon stability. Future studies should prioritize deep soil carbon dynamics, the interactions of carbon, nitrogen, and phosphorus cycles under warming scenarios, and the underlying biological mechanisms behind these responses. Furthermore, the integration of long-term warming experiments with Earth system models is essential for reducing the uncertainties of model predictions regarding future carbon-climate feedback in these climate-sensitive ecosystems.

## INTRODUCTION

Anthropogenic activities have dramatically increased atmospheric greenhouse gas (GHG) concentrations since the mid-20th century, triggering global warming [1]. The global land surface has been warmed by ∼1.6°C over the period 2011–2020 compared to that of 1850–1990 [2]. The elevated temperature has profound effects on terrestrial carbon cycling by affecting both plant carbon uptake and carbon emissions from microbial decomposition, thereby altering the trajectory of terrestrial carbon-climate feedback [3,4]. As a climate change-sensitive region, the Tibetan Plateau has experienced intensive warming over recent decades (0.2–0.7°C per decade; [5]), with rates being more than twice the global average [6]. This substantial climate warming poses significant changes in ecosystem carbon cycling on the plateau, primarily composed of alpine ecosystems with 67% of the areas underlain by permafrost (ground that remains completely frozen for at least two years straight) [7–9]. Particularly, high soil carbon stocks (15.3–21.7 Pg C in the top 3 m; 1 Pg = 10^15^ g; [9,10]) and slow rates of soil microbial decomposition render the ecosystem carbon cycle in this unique geographic unit highly susceptible to climate change [11]. Unraveling the impacts of elevated temperature on ecosystem carbon cycling on the Tibetan Plateau is therefore crucial for elucidating terrestrial carbon-climate feedback at regional, national, and even global scales.

Alpine grasslands cover nearly 70% of the Tibetan Plateau and consist mainly of two major types: alpine steppe and alpine meadow, covering 36% and 32% of the area, respectively [7,11]. This area also scatters swamp meadow in highly wet environments [9]. Over the past few decades, extensive efforts have been directed towards simulating warmer conditions in alpine grasslands on the Tibetan Plateau (‘Tibetan alpine grassland’ hereafter). A variety of methods have been used, encompassing *in-situ* warming experiments [12,13], observation or soil transplantation along altitude/temperature gradients [14], and laboratory incubations [15]. Among these approaches, *in-situ* manipulative experiments are the optimum choice, as they provide more robust cause-effect links and higher ecological relevance than others [16].

**Figure 1.**
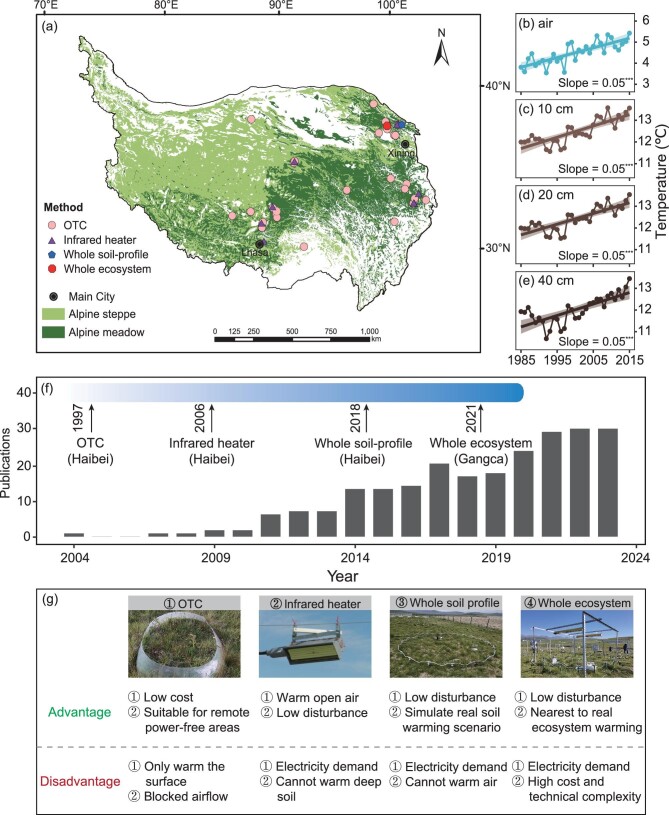
Overview of climate warming trends and *in-situ* warming experiments in the Tibetan alpine grasslands. Locations of the *in-situ* warming experiments (*n* = 57) conducted in alpine grasslands on the Tibetan Plateau (a), and the variation in mean annual air (b) and soil temperature at different depths (c-e) during the period 1985 to 2015. The trend in publications (Web of Science Core Collection) derived from these experiments over the past two decades (f). The advantages and disadvantages of these warming methods (g) are summarized from [19,24]. Data for mean annual air temperature were taken from [188], and those for mean soil temperature were derived from the National Meteorological Information Center of China. The texts and arrows in panel (f) indicate the time when this warming method was first established and its location. Abbreviations: OTC, open top chamber. Significant level: ***, *P* < 0.001 (linear regression). Further details are available in Table S1. The spatial distribution of alpine grasslands was derived from the Vegetation Map of the People's Republic of China (1:1 000 000) (Editorial Committee of Vegetation Map of China 2007). Source of pictures in panel (g): ① [189]; ② offered by S. Wang; ③ [122]; ④ taken by Y. Bai. Review drawing number: GS 京 (2024)2626号

In this regard, the first *in-situ* warming experiment, utilizing an open top chamber (OTC), was initiated in 1997 in an alpine meadow and successfully heated both the air and topsoil [12]. Following this first trial, OTCs were gradually installed in other types of alpine grassland (i.e. alpine steppe and swamp meadow) [17,18] (Fig. 1a. However, the efficacy of OTCs is limited by their passive warming approach, which can create issues such as asymmetrical heating (e.g. the temperature rises more during daytime than at nighttime) and blocked airflow [19]. Bearing this limitation in mind, active warming techniques (i.e. infrared heaters) have been introduced [20,21]. However, this active top-down heat transfer approach struggles to simulate deep soil warming [22], which is predicted to follow air temperature trends [23] (Fig. 1b–e). To address this deficiency, experiments involving whole soil-profile warming, capable of heating deep soils, and whole ecosystem warming, simulating the warming of air together with the whole soil profile, have been conducted in an alpine meadow [24] and a swamp meadow, respectively [25] (Fig. 1a). Nevertheless, constrained by power requirements and technical challenges, these two types of warming experiments have not yet been widely implemented. *In-situ* warming experiments have been constantly evolving over the past two decades, and there has been a great deal of progressive exploration of the responses of alpine grasslands to climate warming and the underlying mechanisms (Fig. 1f–g). Against this background, it is timely to provide a synthesis of the advances made so far, to help progress research in this field to the next level.

This review presents an in-depth overview of current knowledge about the effects of *in-situ* warming on ecosystem carbon cycling. We aim to provide ecological insights into the potential mechanisms involved, including both abiotic (e.g. soil water and nutrients status) and biotic drivers (e.g. plant and soil microorganisms). Our objectives are threefold: (1) to explore how *in-situ* warming alters abiotic and biotic attributes that could affect ecosystem carbon cycling, (2) to elucidate how these factors mediate warming-induced changes in carbon cycling, and (3) to outline critical knowledge gaps and identify emerging research imperatives for future endeavors. By doing so, we anticipate that our review will shed light on the mechanisms behind the responses of the ecosystem carbon cycle to climatic warming in this climatologically vulnerable region.

## WARMING EFFECTS ON ABIOTIC AND BIOTIC ATTRIBUTES

Soil water and nutrient availability, alongside plant and soil microbial characteristics, serve as pivotal regulators of the terrestrial carbon cycle [4,26–28]. As the climate warms, these factors are expected to undergo significant changes, thereby affecting ecosystem carbon cycling processes [29]. In this section, we concisely review warming effects on these abiotic (e.g. soil moisture and nutrient availability) and biotic variables (e.g. plant community structure, phenology, functional traits, and soil microbial attributes).

### Soil hydrological and nutrient status

**Figure 2.**
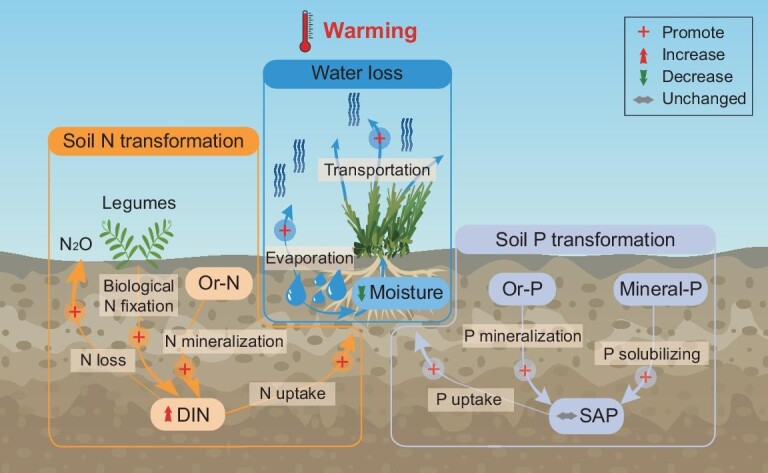
Schematic diagram of the impacts of experimental warming on soil water loss and nutrient transformation in Tibetan alpine grasslands. Warming accelerates soil nutrient transformation processes and enhances their utilization by organisms. Meanwhile, it also increases plant transportation and soil water evaporation, resulting in a decline in soil moisture. Abbreviations: Or-N, soil organic nitrogen; DIN, dissolved inorganic nitrogen; Or-P, soil organic phosphorus; Mineral-P, mineral-associated inorganic phosphorus; SAP, soil available phosphorus. Symbols in the diagram: the red cross denotes the promotion of water and nutrient cycling processes; the red arrow indicates an increase in nutrient or water levels, the green one denotes a decrease, while the grey one means no change. The plant images were derived from 58pic.com.

Soil moisture is a key factor determining the response of the terrestrial carbon cycle to climate warming [28]. Most of the field warming experiments in alpine grasslands reveal that warming substantially increases topsoil temperature, by 0.3–3.0°C, but decreases soil moisture by 2.8%–36.0% [30] (Table S1). This reduction in soil moisture has been mainly attributed to elevated temperature, which, coupled with a substantial water-air pressure difference, enhances plant transpiration by promoting water diffusion out of the leaves [31]. Concurrently, soil warming increases saturation vapor pressure, exacerbating the vapor pressure deficit and thereby strengthening soil water evaporation [32]. The combined enhancement of these two processes promotes ecosystem evapotranspiration, leading to topsoil drying [21] (Fig. 2). Notably, a warming-induced increase in plant transpiration (42.2%–48.1%) was found to be much stronger than that in soil water evaporation (16.2%–18.6%). This phenomenon could be attributed to the direct promotion of transpiration by higher temperature, coupled with the indirect stimulation of plant-induced water dissipation caused by an extended period of vegetation activity under warmer conditions [21]. By contrast, the wetter swamp meadows, especially those in permafrost-affected regions, exhibit stable soil moisture levels following experimental warming, despite increased evapotranspiration [33,34]. This stability is possibly due to lower evapotranspiration compare to precipitation, and substantial soil water storage (volumetric moisture >40%) enables the rapid replenishment of water loss [25,35]. In these ecosystems, warming-driven earlier soil thawing also allows higher soil water availability in early spring, potentially influencing vegetation green-up and GHG emissions during this critical period [25,33].

Soil nutrient availability, especially for nitrogen, is acknowledged as a critical determinant of plant productivity and microbial decomposition, which substantially modulate ecosystem carbon balance in response to climate change [36–38]. In Tibetan alpine grasslands, experimental warming has led to a slight increase in topsoil nitrogen availability, primarily in the form of ammonium (NH_4_^+^-N) [39]. The enrichment of soil dissolved inorganic nitrogen (DIN) under warmer conditions reflects a nuanced balance among multiple nitrogen input and output processes. In regard of the soil DIN gain processes: soil net nitrogen mineralization rate substantially increases under warming conditions [40,41], implying elevated soil nitrogen supply as a result of warming. Moreover, long-term warming may promote the flourishing of legumes, facilitating plant-microbial symbiotic nitrogen fixation and further enriching soil nitrogen availability [42]. In respect of the loss processes: warming elevates plant DIN consumption and promotes nitrogen relocation from soil to plants [43], alongside increased nitrous oxide emission [44,45]. However, soil DIN gain processes outweigh the losses, as evidenced by a 29% increase in NH_4_^+^-N [39] (Fig. 2). According to the above synthesis, experimental warming accelerates certain nitrogen-cycling processes, leading to a more rapid nitrogen cycle in these alpine grasslands: a trend which resonates with observations in cold ecosystems globally [46].

In contrast to soil DIN, soil available phosphorus (SAP) remains largely unchanged under experimental warming, although an acceleration of organic phosphorus mineralization [47,48] and inorganic phosphorus solubilization has been observed [49]. This apparent contradiction may be explained by an increased demand for phosphorus from both plants and soil microbes, leading to the assimilation of SAP generated from the transformation of organic and inorganic phosphorus sources [49]. Furthermore, soil warming may also enhance SAP leaching by accelerating water and heat exchanges across soil layers, a process which is potentially relatively pronounced in permafrost-underlain alpine grasslands with high soil moisture [50]. Therefore, while experimental warming can promote the transformation of soil phosphorus into an available state, high plant and microbial uptake may offset such changes, leaving SAP unchanged (Fig. 2). Collectively, soil nitrogen and phosphorus transformations in Tibetan alpine grasslands were visibly stimulated under warmer scenarios (Fig. 2). The expedited release of soil available nutrients, utilized by plants for their growth, as well as by soil microbes for organic matter formation and decomposition [51], plays a vital role in mediating ecosystem carbon balance under warming scenarios.

### Plant community structure

**Figure 3.**
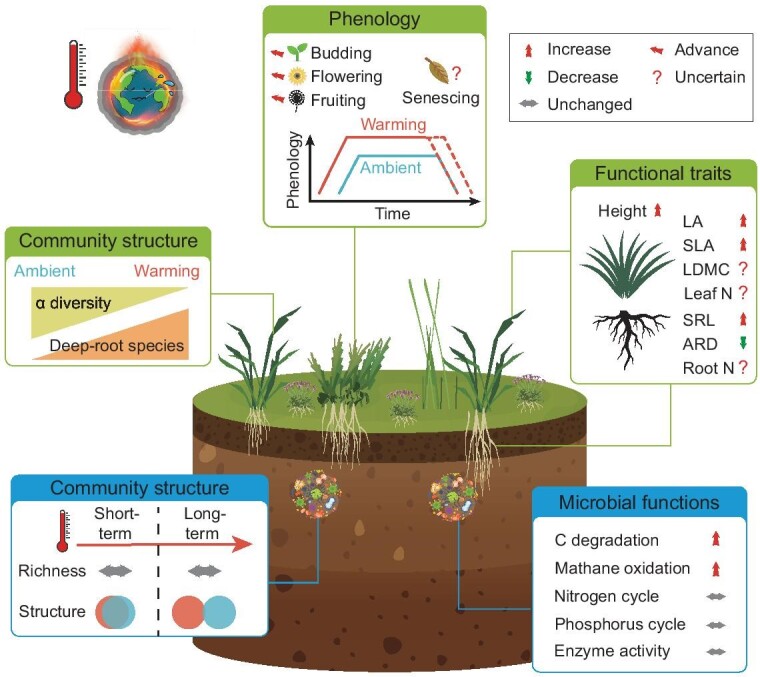
A schematic illustration of the influence of experimental warming on plant and microbial community attributes in Tibetan alpine grasslands. Warming leads to a decrease in plant species richness, an alteration in community structure, an extension of the growing season, and changes in morphological traits of foliage and root. The soil microbial community structure exhibits significant change only with long-term warming, without affecting species richness. Functional genes related to carbon cycling are more abundant in warmed conditions, whereas those related to nitrogen and phosphorus cycling remain unchanged. Soil extracellular enzyme activity is insensitive to warming. Abbreviations: LA, leaf area; SLA, specific leaf area; LDMC, leaf dry mass content; SRL, specific root length; ARD, average root diameter. Symbols in the diagram: the red arrow represents an increase in plant and soil microbial attributes, the green one denotes a decrease, while the grey one means no change; the question mark signifies uncertainty in the process in response to warming. The plant images were derived from 58pic.com.

Plant community is pivotal in regulating terrestrial material cycles (e.g. ecosystem carbon cycling) and energy flow [27]. Experimental warming has been shown to induce shifts in vascular plant community structure, characterized by a notable change in species composition and a reduction in species diversity across Tibetan alpine grasslands (Fig. 3). Specifically, warming often accompanies a decline in the relative abundance of shallow-rooted species (e.g. non-leguminous forbs) and an increase in the prevalence of deep-rooted species (e.g. graminoids) [18,52]. These asynchronous shifts may partly result from topsoil drying (discussed in Section ‘Soil hydrological and nutrient status’), which exacerbates water stress for shallow-rooted plants with limited water absorption plasticity [52]. This enhanced stress intensifies competition for water and drives hydrological niche differentiation, serving as an important causative factor for restructuring the plant community [53]. Besides, warming-enhanced soil nutrient availability may also favor species with specific nitrogen utilization strategies (e.g. preference for NH_4_^+^-N or NO_3_^–^N), further reshaping community composition [54]. Additionally, an increase in legume cover under long-term warming has been observed in some alpine grasslands as mentioned above [42,55]. This increase could be attributed to the reduced soil water content (e.g. some legumes belonging to deep-rooted species; [18]) and accelerated soil phosphorus transformation rates (i.e. a high phosphorus demand for legumes) [56]. These changes in plant community structure are expected to have profound effects on ecosystem stability and carbon-related functions, such as carbon uptake [42].

In addition to changes in plant community composition, species richness in alpine grasslands is generally reported to decline with warming [57] (Fig. 3). Several potential mechanisms may drive this reduction: (1) Warming often triggers water deficit in the topsoil (discussed in Section ‘Soil hydrological and nutrient status’), creating an environmental filter that selectively disadvantages species intolerant to drought stress and with poor water-use plasticity [58]. (2) Warming may further expand the ecological niche of dominant species, due to their superior adaptability, which enhances competitive exclusion and thus reduces species diversity [59]. (3) Increased plant height under warming conditions can intensify the shading effects of high-stature species over low-stature ones, magnifying size-asymmetric competition for light and promoting the loss of dwarf species [60]. The warming-induced decline in species diversity observed in Tibetan alpine grasslands was consistent with those findings derived from some European alpine regions, signaling a potential threat to alpine biodiversity under future climate warming scenarios [61]. While the overall magnitude of these effects appears relatively modest (reducing plant richness by 12.2% and Shannon-Wiener index by 6.3%; [57]), the impacts of such slight changes on ecosystem functioning, such as carbon sequestration, cannot be ignored. The loss of certain functionally unique species could reduce the resilience and stability of plant communities to environmental changes, potentially disrupting carbon cycling processes [42]. Furthermore, shifts towards dominance by a few competitive species might lead to homogenized plant-sourced carbon inputs, possibly altering the dynamics of both soil organic carbon (SOC) and its fractions [62].

Besides vascular plants, mosses, a form of cryptogam widespread in Tibetan alpine grasslands (e.g. *Didymodon* spp. and *Sphagnum* spp.) [63], also played a crucial role in moderating ecosystem carbon sequestration [64]. While climate warming has been shown to reduce moss dominance at high latitudes [65], the response of mosses to warming in the equivalently cold alpine grasslands and their impacts on ecosystem carbon uptake need further elucidation. More importantly, one issue requiring some attention is that *in-situ* warming experiments may not fully capture the dynamics of natural plant communities over time. For example, a rapid increase in temperature in the warming experiments may not accurately simulate the gradual and continuous warming experienced in natural settings [66]. There might be a lag in species mortality and replacement with the effects of species interactions, as well as a limited presence of propagules for species adapted to progressively warmer conditions, delaying true plant community shifts [67,68]. As a result, despite the fact that *in-situ* experimental warming provides valuable insights into potential trends, the related findings should be integrated with long-term observations, transplant studies, or ecological models to better account for the complex changes in plant communities with climate change [68–70].

### Vegetation phenology

Climate warming markedly affects vegetation phenology (i.e. green up and withering), thereby regulating carbon assimilation during the period of vegetation growth [71]. In alpine grasslands, the majority of *in-situ* warming experiments have shown that an elevated temperature advances the start of vegetation activity (e.g. advancing first leafing out by 1.7–9.2 days; Fig. 3). This advancement may be due to the fact that a reduction in the time required for plants to meet the cumulative heat demands of a particular phenological event under warmer conditions [72]. Additionally, earlier snowmelt and soil thawing, particularly in permafrost-affected alpine grasslands, facilitate the availability of water and nutrients in early spring, further contributing to earlier plant emergence [25,73].

In contrast to the consensus on the advancement of the green-up period, the effects of warming on the vegetation withering period remain uncertain (e.g. unchanged or delayed in completely withering; Fig. 3). This uncertainty may be determined by the plant's trade-offs between carbon optimization and nutrient metabolism [74]. Specifically, an unchanged foliage senescence period may hinder plants from reaching their carbon uptake saturation, preventing competition for resources throughout the entire life cycle; conversely, delayed senescence would diminish nutrient resorption due to the effect of frost [75]. In terms of environmental factors, the variation of foliage senescence time has also been shown to depend on warming-induced changes in soil moisture and leaf temperature [72,76]. For instance, seasonal soil dryness can slow photosynthetic product transport, delaying foliage senescence [77]. Contrariwise, high air and leaf temperature may prompt early foliage senescence due to petiole hydraulic exhaustion or leaf cell metabolic disorders [78,79]. The interplay between the plant's internal requirements and environmental changes jointly modulates the withering time under climate warming scenarios. It can be speculated that warming may delay foliage senescence if plants have not fully saturated their carbon uptake or if seasonal soil drought persists. In contrast, if plants adopt conservative nutrient resorption strategies (e.g. tend to recycle earlier; [80]) or endure leaf heatwaves, such a prolongation effect may be neutralized, leading to unchanged phenology. Despite the inconsistent responses in foliage senescence timing, the overall length of the vegetation growing season has been substantially extended under warming conditions [72]. Such an extension of the photosynthetic period is expected to enhance the ecosystem carbon sequestration [73,81].

### Plant functional traits

Plant functional traits, such as morphological and chemical traits, exhibit substantial plasticity in response to elevated temperature, subsequently influencing plant community assembly and ecosystem carbon balance [82]. At the species level, there has not yet been a consensus on the warming effects on leaf and root morphological traits, largely due to the paucity of data and species-specific responses (Fig. 3). However, a general trend has emerged, indicating increases in height, leaf area, together with specific leaf area as the environment becomes warmer [41,55] (Fig. 3). These changes in leaf functional traits would assist plants in accessing light resources, enhancing photosynthesis and contributing to biomass accumulation [83]. Conversely, no consistency has been observed in the response of leaf dry matter content to experimental warming (Fig. 3). This inconsistency might be related to the wide range of soil moisture reduction observed with experimental warming (ranging from 2.8%–36.0% as mentioned in Section ‘Soil hydrological and nutrient status’). Specifically, if soil moisture drops below the threshold of drought stress for plants, they may adopt more stress-tolerant strategies (e.g. higher leaf dry matter content), and vice versa [84]. Regarding root morphological traits, specific root length tends to increase under warming conditions, whereas average root diameter decreases (Fig. 3). These responses are coherent with their positions as negative covariates on the root economics spectrum [85], suggesting a shift in plant resource uptake strategies towards strengthening their own uptake capacity (do-it-yourself strategy) [86].

For chemical traits, both leaf and root nitrogen concentrations show species-specific responses to experimental warming (Fig. 3). Leaf nitrogen concentration generally decreases or remains unchanged under warming [41,55]. This inconsistency may be associated with greater stature, larger leaf areas, and the resultant higher aboveground growth following warming (see Section ‘Ecosystem productivity and plant biomass’ for a detailed description), as the enhanced biomass can dilute the nitrogen concentration in tissues, leading to decreased or stable nitrogen concentration. Likewise, root nitrogen concentration shows contrasting responses to warming depending on the species (i.e. increased or decreased; [40,41]). Increased root nitrogen concentration is often related to improved soil nutrient availability (discussed in Section ‘Soil hydrological and nutrient status’) and altered root morphological traits under warming conditions. Specifically, the enrichment of soil DIN provides more available nitrogen for uptake, while increased specific root length and decreased average root diameter allow for a higher ability of nitrogen uptake [85,87]. Despite that, warming-induced enhancement in belowground growth may also lead to a growth dilution effect, resulting in a decline in root nitrogen concentration [40].

At the community level, the response of plant functional traits to experimental warming is determined by both intraspecific variation (ITV) and species turnover between treatments [88]. Regrettably, there are insufficient studies in this region to generalize which process predominantly drives community-level trait variation under warming scenarios [41,59]. In a permafrost-affected swamp meadow, community-level functional trait changes were largely determined by ITV [41]; whereas in an alpine meadow, species turnover was the dominant factor [59]. Such differences in dominant processes reflect varying adaptative strategies of species in response to environmental change [88]. When ITV primarily drives community trait variation following warming, it indicates that species adapt to environmental changes through phenotypic plasticity. Conversely, when species turnover dominates, it suggests that environmental changes filter out original species, leading to their replacement by others [89,90]. Overall, from the perspective of functional traits and resource-use strategies, rising temperature is likely to provoke a shift in vascular plants towards aggressive resource acquisition and a high relative growth rate in Tibetan alpine grasslands [41,83]. These variations are expected to influence nutrient uptake and carbon accumulation by plants, thereby regulating the response of ecosystem carbon dynamics to climate warming.

### Soil microbial community structure

Soil microorganisms, being directly involved in soil organic matter decomposition, play a pivotal role as engines driving Earth's biogeochemical cycles [91]. Their responses to climate warming substantially affect soil carbon stability and moderate terrestrial carbon-climate feedback [92]. Soil microbial metabolic activity in Tibetan alpine grasslands is constrained by low temperature, and its response to climate warming is expected to be sensitive [93]. Several studies have reported alterations in microbial species composition, observable as variations in the relative abundance of taxonomic groups at the phylum level (e.g. Acidobacteria; [94]), specific functional groups (e.g. arbuscular mycorrhizal fungi; [95]), or species with contrasting life-history strategies (e.g. copiotrophic *vs.* oligotrophic; [96]). However, the impact of warming on the community structure strongly depends on experimental duration: short-term warming effects (e.g. <3 years) are relatively muted, whereas long-term effects (e.g. ≥3 years) are much stronger [96,97] (Fig. 3). A similar resistance of soil microbes to temperature changes over a short period has also been observed in equally-cold conditions in the Arctic [98], highlighting the potential lag in microbial community structure's change to elevated temperature.

The primary mechanism behind warming-induced changes in microbial community structure lies in variations in the relative importance of stochastic *vs.* deterministic processes governing community assembly [99,100]. As the climate warms, the dominance of stochastic processes is expected to fluctuate substantially, resulting in greater variability and uncertainty in microbially mediated biogeochemical cycling processes [101]. In contrast, deterministic processes gain prominence, underscoring the increasing role of environmental filtering and biotic interactions in driving the microbial community assembly with climate warming [100,102]. For instance, environmental selection mediated by elevated soil temperature might selectively favor the warm-adapted taxa, consequently evolving a distinct community structure compared to the ambient one [103]. Warming-induced changes in soil moisture and substrate availability could also alter the relative abundance of different trophic groups (e.g. drought-tolerance/intolerance or copiotrophic/oligotrophic species), thereby reorganizing microbial communities [96,104]. In the case of biotic interactions, for example, shifts in plant community composition under experimental warming can modify plant-microbe interactions, with alterations in microbial species composition [105]. In addition, climate warming may reshape microbial intra-, inter-, and across-trophic interactions, affecting soil microbial community dynamics [106].

Despite remarkable community structuring, microbial diversity (e.g. species richness and Shannon-Wiener index) tends to remain stable under warming [57] (Fig. 3). This stability is likely because of species reordering within the microbial community, whereby species richness remains constant despite fluctuating proportions of taxonomic groups [107]. Such insurance of the soil microbial species pool, i.e. changing community composition without affecting species richness, preserves diversity at the same high levels as under ambient conditions. This makes soil microbes more resistant to the aggravating environmental stress and the intensified microbial competition upon warming [108].

### Soil microbial functional genes and extracellular enzymes

Shifts in soil microbial community structure under climate warming are likely to influence microbial functional potential, with significant implications for the ecosystem carbon cycle [109,110] (Fig. 3). For example, warming has been observed to be associated with a notable increase in the relative abundance of functional genes for carbon decomposition, such as carbohydrate metabolisms that are denoted as carbohydrate-active enzyme families [110,111]. This shift suggests an enhanced capacity for soil organic matter decomposition, potentially accelerating carbon emissions [112]. In terms of methane metabolism, genes involved in methane oxidation, such as those coding for soluble methane monooxygenase, have shown marked increases; while genes associated with methane methanogenesis remain largely unchanged [110]. These functional gene shifts align with observed changes in methane fluxes under warming (see Section ‘Carbon release’ for a detailed description; [113]). However, literature regarding functional genes involved in nitrogen and phosphorus cycles are scant, with minimal effects of warming being reported [49,114]. Despite this, some studies have noted that warming intensifies certain soil nitrogen and phosphorus transformations ([40, 41] for nitrogen; [47,48] for phosphorus). Such discrepancy underscores the need for further studies to integrate warming effects on soil nutrient transformations and their associated microbial mechanisms.

Beyond functional genes, soil extracellular enzyme (SEE) plays a more direct role in regulating soil carbon and nutrient cycling processes, ranging from the decomposition of soil organic matter to the mineralization of soil nutrients [115,116]. These enzymes, stabilized in the soil matrix (e.g. forming stable complexes with humus, clay, or their mixes), are decoupled from living cells and highly sensitive to changes in the soil environment [117]. However, a recent meta-analysis has shown that no significant effects of experimental warming on the activity of SEEs involved in soil carbon, nitrogen, and phosphorus cycling were observed across Tibetan alpine grasslands [118], consistent with global trends [119,120]. One possible explanation for the lack of significant response is that while elevated temperature may directly enhance SEE activity, occurrent soil drying could negate this promoting effect [121]. Nevertheless, this point cannot fully interpret the unresponsiveness of SEE in Tibetan alpine grasslands, since enzyme activities remain steady even under long-term warming and precipitation-supplemented conditions [118].

Two possibilities for the above-mentioned situation are the magnitude and duration of experimental warming, both of which are recognized as critical factors [119,120]. In most studies across Tibetan alpine grasslands, soil temperature was increased by a modest degree (1–2°C), resulting in unchanged SEE activity [118]. Conversely, hydrolytic enzymes like β-1,4-glucosidase and acid phosphatase showed a significant increase in activity for surface soils after experiencing three years of high-level warming at ∼4°C [122]. This suggests that the intensity of warming may indeed be a key determinant of SEE activity in response to warming in alpine grasslands. Moreover, as discussed in previous Sections, plant community structure, soil microbial composition and their functions remain stable during short-term warming, but may gradually alter with prolonged warming. These ongoing changes might render SEE activity to remain unresponsive initially but enhance over time under sustained warming [116]. Besides, long-term warming also alters the chemical fractions of SOC (e.g. ratio of recalcitrant and labile carbon; [123]), coupled with shifts in enzymatic functions, may eventually lead to substantial carbon loss over time [124,125].

## CARBON CYCLES IN RESPONSE TO EXPERIMENTAL WARMING

Climate warming can affect both carbon uptake and release in terrestrial ecosystems, altering the global carbon cycle and its feedback to climate change [3]. A comprehensive understanding of these responses and the corresponding mechanisms is crucial for predicting ecosystem carbon balance under future climate scenarios. In the following section, we briefly review the effects of *in-situ* experimental warming on the ecosystem carbon cycle in Tibetan alpine grasslands, focusing on key aspects such as plant carbon uptake (e.g. plant productivity), litter decomposition, soil carbon emission (e.g. carbon dioxide [CO_2_] and methane fluxes), ecosystem carbon balance, and fates of soil carbon stock and its fractions.

### Ecosystem productivity and plant biomass

**Figure 4.**
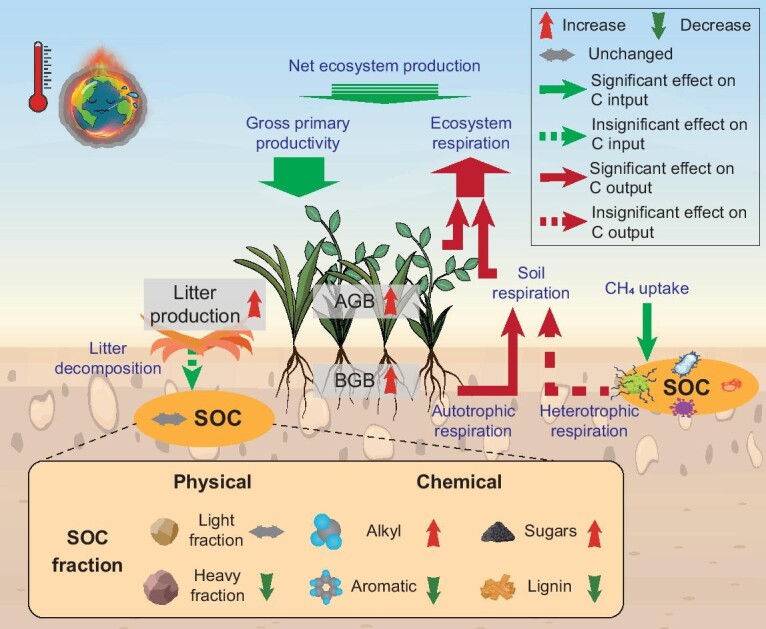
Diagrammatic representation showing the effects of *in-situ* warming on ecosystem carbon cycle in Tibetan alpine grasslands. Warming substantially enhances ecosystem carbon sequestration, resulting in an accumulation of plant biomass and litter production. Most of the carbon release processes were intensified except soil heterotrophic respiration. While soil organic carbon stocks show no overall change, warming markedly alters their fractions. Abbreviations: AGB, aboveground biomass; BGB, belowground biomass; SOC, soil organic carbon. Symbols in the diagram: the red arrow represents an increase in SOC fractions and plant biomass, the green one denotes a decrease, while the grey one indicates no change. The red arrow with line indicates carbon output processes, while the green arrow with line represents carbon input processes; a solid line denotes a significant effect of warming, while a dashed one shows an insignificant effect. The plant and SOC fraction images were derived from 58pic.com.

Gross primary productivity (GPP) is the crucial first step in CO_2_ intake into the ecosystem [31]. In Tibetan alpine grasslands, GPP is estimated to be 122.4–872.1 g C m^−2^ during the growing season (generally May to September) [126], with a majority of the literature reporting an enhancement upon experimental warming [44,127] (Fig. 4). This increase in GPP could be directly linked to higher leaf area index (LAI) and leaf photosynthesis-related parameters upon warming [31,128]. For the former, increased LAI expands the space available for carbon exchange between foliage and the atmosphere, improving both the proportion of diffuse canopy interception and direct canopy light interception [129]. Regarding the latter, an elevation in temperature would directly promote photosynthesis, as the current average daily maximum temperature during the growing in this region (12.2°C; 95% CI: 12.1–12.3) falls below the optimal temperature for plant productivity (14.7°C; 95% CI: 14.6–14.8) [129]. Warming has also been shown to enhance stomatal conductance, chlorophyll content, soluble sugar, peroxidase, etc., further promoting net photosynthesis, and thus GPP, through optimizing light utilization [130]. Although GPP has been found to be generally boosted by warming, the magnitude of the increase varied across seasons in specific alpine ecosystems. For instance, in a swamp meadow, warming enhanced carbon sequestration before the period of peak plant growth (i.e. maximum GPP). However, this positive effect vanished post-peak, largely owing to a decline in foliage nitrogen and phosphorus concentration [34].

In most *in-situ* warming experiments across Tibetan alpine grasslands, elevated temperature led to an increased plant carbon accumulation, as indicated by the growth in both above- and below-ground biomass [30,39] (AGB and BGB hereafter; Fig. 4). For AGB, this warming-induced enhancement can be attributed to several key factors: (1) In such cold ecosystems, experimental warming may mitigate the temperature constraints on vegetation growth, directly promoting growth rates and biomass accumulation [131]. (2) Species would respond positively to the improved soil nitrogen availability under warmer conditions, resulting in increased aboveground productivity [13]. (3) The prolongation of vegetative phenology allows for a longer period of vegetation carbon sequestration, further contributing to AGB accumulation [72,73]. (4) In warmer environments, species exhibit taller stature and larger leaf area, which helps plants access more light and enhances photosynthetic efficiency, contributing to biomass accumulation [132]. (5) Changes in root morphological traits and nutrient utilization strategies upon warming facilitate more more efficient nutrient uptake and utilization, thereby supporting increased AGB [54].

Similar to AGB, the gains in BGB under warming scenarios are also largely determined by the alleviation of temperature and nutrient limitations [133]. Moreover, warming-induced soil moisture reduction further promotes the accumulation of BGB [134]. Specifically, species with deeper root horizons would tend to thrive in the community due to their ability to access water from deep soil layers to counter the deficit in the topsoil, ultimately increasing root biomass [52]. Warming can also intensify plant nutrient requirements. In some cases, to adapt to the new conditions, plants have altered root morphological traits (e.g. higher specific root length) and invested more biomass in fine roots to enhance nutrient accessibility [135]. In particular, in permafrost-affected alpine grasslands, climate warming could lead to an extension of belowground phenology (e.g. phenology of roots) due to a combination of earlier soil thaw and delayed freezing, further promoting BGB [25,134]. An increase in plant biomass would have a dual effect on SOC dynamics: it contributes to SOC accumulation via litter decomposition and rhizodeposition, and potentially stimulates SOC loss through the rhizosphere priming effect [53,136]. These complex interactions demonstrate the crucial role of plant biomass in moderating ecosystem carbon balance under climate warming.

### Litter production and decomposition

Litter serves as a crucial conduit for carbon and nutrient transfer from plants to soil, driving the formation of soil organic matter [135,137]. In grassland ecosystems, litter production is often assumed to be approximately equal to net primary productivity (NPP) under steady-state conditions [138]. On this basis, litter production in alpine steppe and alpine meadow has been estimated at 66.9 (8.3–126.6) g C m^−2^ year^−1^ and 179.1 (11.8–469.2) g C m^−2^ year^−1^, respectively, using NPP data from 2001 to 2010 (expressed as mean [lower extreme, upper extreme]; [139]). While empirical data on the effects of *in-situ* warming on litter production are scarce, the observed increase in ANPP and BNPP under warming suggests that litter production is theoretically expected to rise as well [138]. Despite its crucial role as a carbon and nutrient reservoir, litter is often overlooked when measuring ecosystem carbon balance [140]. In addition, accumulated litter on the surface may also serve as a mechanical barrier to modify soil temperature, moisture, as well as vegetation dynamics [141].

Litter decomposition plays a vital role in restocking soil carbon and nutrient pools [137]. However, the effects of warming on litter decomposition remain ambiguous across Tibetan alpine grasslands, with studies reporting both promotion and inhibition (Fig. 4). These inconclusive observations align with the tendencies found in global biomes, for which the overall effect of warming on litter decomposition remains uncertain [142]. These mixed results likely arise from complex interactions between warming-induced changes in soil environments (e.g. temperature and moisture), shifts in litter quality, as well as differences in experimental methodology (e.g. OTC and others) [143,144]. Specifically, on the one hand, elevated soil temperature directly enhances the activity of decomposers, such as enzymes and specific saprophytic fungi that physically break down the litter mass, leading to faster litter decomposition [145]. Moreover, it may also favor the activity of fauna and prolong its active period, contributing to accelerated litter turnover [146]. On the other hand, warming-induced changes in soil environments and leaf chemistry may counteract the decomposition-promoting effects of higher temperature. For instance, soil and litter drying under warming can slow decomposition rates [147]. In addition, a warming-induced increase in leaf nitrogen resorption can lower nitrogen concentration in leaf litter, rendering it more resistant to microbial decomposition [148].

Notably, the differences in methodology used for simulating climate warming may further complicate the outcome of litter decomposition [143]. Experiments using OTCs have often reported decreased litter decomposition rates, whereas active warming techniques, such as infrared heaters and soil cables, have demonstrated enhanced decomposition [143,144]. These discrepancies are largely attributable to the varying magnitude of warming effects on soil water conditions [147]. Given that the contrasting responses may be attributed to warming methodology, further explorations using more rigorous methods are highly needed to elucidate the response of litter decomposition to climate warming across Tibetan alpine grasslands.

### Carbon release

Carbon release, including ecosystem respiration (Reco), soil respiration (Rs), and methane fluxes, represents a critical pathway for carbon output from terrestrial ecosystems to the atmosphere [31]. A recent data synthesis showed that Reco in alpine grasslands ranged from 62.1 to 627.3 g C m^−2^ during the growing season, which was increased by ∼15% under experimental warming [126,149] (95% CI: 7%–24%; Fig. 4). This enhancement is primarily driven by plant-derived fluxes, comprising aboveground plant respiration and root respiration, e.g. the latter of which increased by 31% [95% CI: 8%–59%] 149]. In contrast, the soil-derived flux (i.e. soil heterotrophic respiration; Rh), stemming from microbial decomposition of organic matter, remained largely unaffected by experimental warming [149].

Interestingly, although elevated temperature is generally expected to boost microbial decomposition and soil CO_2_ efflux, the response of Rh is more complex in the real world. Factors such as soil drying, reduction in soil microbial biomass and microbial thermal acclimation under warming scenarios might counterbalance the expected promotion, resulting in an overall neutral Rh response [150]. The enhanced plant respiration can be offset by increased vegetation productivity, while stable Rh does not result in greater losses of soil carbon. Such diverse responses may contribute to a relatively stable soil organic carbon pool under warming conditions, preventing an amplification of the carbon-climate feedback [151]. Similar to soil CO_2_ flux, the influence of experimental warming on methane fluxes in Tibetan alpine grasslands is contingent upon soil water conditions. In the case of relatively wet swamp meadows, warming has been found not to alter methane emission, likely resulting from the unchanged water-filled pore space and methanogen abundance [45,113]. Conversely, in relatively dry alpine steppe and meadow ecosystems, experimental warming has been shown to enhance methane uptake by increasing the abundance of methanotrophs [110]. Despite these divergent responses, the overall increase in methane uptake may strengthen the ecosystem's capacity as a methane sink in this region [57,113] (Fig. 4).

### Net ecosystem carbon balance

The GPP usually exceeds Reco across Tibetan alpine grasslands, indicating overall positive values for net ecosystem production (NEP) during the growing season (NEP = GPP-Reco; [31,152]). The NEP is estimated as 23.0 ± 13.8 g C m^−2^ year^−1^ in the alpine steppe and 36.7 ± 4.6 g C m^−2^ year^−1^ in the alpine meadow (expressed as mean ± se; [149,152]). Under warming conditions, the trend of NEP remains largely unchanged in most alpine grasslands, with the exception of particularly wet areas. Specifically, in the relatively dry alpine steppe and meadow ecosystems, the close increments of both GPP and Reco result in little change in NEP [153]. While in highly wet swamp meadows, experimental warming enhanced NEP, greatly strengthening ecosystem carbon sink [34]. These divergent responses in NEP, modulated by soil water conditions, align with the global analysis that warming tends to promote net carbon sequestration in humid ecosystems but not in arid environments [154]. Nevertheless, the variations of NEP obtained from *in-situ* warming experiments seem to conflict with long-term eddy covariance observations, which show an overall enhancement of ecosystem carbon sink with climate change in this region [152]. This apparent discrepancy may arise from differences between simulative and actual environmental changes that have occurred. Experimental warming primarily led to temperature increases and a concurrent decline in soil moisture (Section ‘Soil hydrological and nutrient status’), but real-world scenarios often involve temperature elevation, CO_2_ enrichment, and changes in precipitation regime in this region [6]. The interplay of these climate change factors might offset the diminished NEP observed in arid environments, which may contribute to the long-term positive trends in NEP [152]. This insight highlights the importance of considering the comprehensive effects of climate change, beyond temperature alone, to fully understand ecosystem responses.

### Soil carbon stock and its fractions

Owing to the restrictions placed on plant growth and soil microbial decomposition by the cold environment, vegetation carbon stock tends to be relatively small, rendering soil the predominant component of ecosystem carbon stock in Tibetan alpine grasslands [131]. The average SOC density (carbon amount per area) at 1 m depth ranges from 6.5 to 20.6 kg C m^−2^ [9,155,156]. Interestingly, experimental warming appears not to affect SOC stock (Fig. 4), regardless of the warming magnitude, duration, or size of the standing SOC stock [123,149]. This neutral response is consistent with findings from a meta-analysis of 143 *in-situ* warming experiments worldwide [157]. However, this lack of change seems to be inconsistent with the decadal soil carbon accumulation reported from a regional-scale resampling at 10-year intervals [158].

One plausible explanation to reconcile the above-mentioned inconsistency is the high spatial heterogeneity of soil carbon pools, which, coupled with a minimal number of replicates and the limitations of sample size in the *in-situ* warming experiments may obscure detectable changes in SOC content across this unique geographic unit [159]. To address these challenges and improve the generalizability of findings, future studies could increase the number of true replicates at individual sites to mitigate small-scale spatial heterogeneity [160]. Moreover, expanding the size of warming experiments, particularly by adopting a networked approach, would enable a more holistic understanding and consistent response across regional scales [161]. A second possibility is that the observed increase in carbon accumulation was likely to be allocated more to plant biomass, resulting in more detectable changes in plant carbon pool rather than soil carbon pool after short-term warming (Section ‘Ecosystem productivity and plant biomass’). Combined with the concomitant accelerating dissolved organic carbon transport to deep soils upon warming, these phenomena could jointly account for the unremarkable rise in soil carbon stock [39]. Third, as mentioned above, real-world environmental changes in this region involve multiple factors, including climate warming, CO_2_ enrichment, nitrogen deposition, and humidification, etc. [6,11]. However, *in-situ* experimental warming has primarily proved effective in simulating temperature elevation and consequent changes in related environmental parameters, such as soil moisture (Section ‘Soil hydrological and nutrient status’). These discrepancies between simulated environmental changes in experimental manipulation and actual environmental changes may account for the inconsistent changes in soil carbon stock between *in-situ* warming experiments and decadal-scale resampling [29].

Whilst changes in carbon stock have not been detected under experimental warming, significant variations in soil carbon fractions (e.g. physical fraction and chemical composition) have been demonstrated across Tibetan alpine grasslands [111,123,159,162] (Fig. 4). As an illustration, a 20-year warming in an alpine meadow decreased the heavy fraction of soil carbon without altering the light fraction. The decline in heavy fraction of soil carbon is likely due to the diminished inputs from microbial necromass and the subsequent suppression of the microbial pathway of soil carbon formation. The unchanged light fraction is likely due to a balance between increased litter input and enhanced decomposition of light fraction [123]. Similarly, another study in a swamp meadow observed a stable light fraction of soil carbon upon warming [111]. However, this study also identified an increased proportion of mineral-associated organic carbon, largely due to the microbial tendency to decompose plant-derived carbon, leading to the accumulation of microbial-derived carbon under warming conditions [111].

The response of SOC chemical composition to warming varied by grassland type: in an alpine meadow, the proportion of carboxylic C decreased but O-alkyl C increased; in a swamp meadow, the percentage of alkyl C increased whereas that of aromatic C declined [123,159]. Such contrasting responses may be driven by ecosystem-dependent differences in plant composition and soil microbial community, where plant composition determines the quality of carbon inputs, and soil microbes influence substrate decomposition performances [163,164]. Specifically, the taxa and decomposability of plants differed across alpine grasslands, creating heterogeneity in the chemical composition and degradability of SOC [165]. Similarly, the composition of soil microbial communities and their response to warming might also vary among ecosystem types, affecting microbial carbon substrate-uptake preferences and, eventually, the chemical composition of SOC responses to experimental warming [95,166]. Overall, given the variations in source, decomposability, and residence time of different SOC fractions, changes in these factors will inevitably affect SOC stability, potentially moderating carbon-climate feedbacks in these alpine grasslands [167].

## ENVISIONING THE FUTURE

Over the past two decades, substantial progress has been made in understanding ecosystem carbon cycle responses to warming across Tibetan alpine grasslands, accompanied by advancements in experimental technologies (Fig. 1f–g). Warming has been shown to shift vegetation structure and extend the growing season, enhancing ecosystem carbon uptake. Meanwhile, warming alters soil microbial communities and their functions related to carbon cycling, indicating an increased potential for SOC decomposition. However, the impact of warming on vegetation carbon sequestration is much stronger than that on soil carbon release. No significant alterations in the soil carbon pool were observed, but remarkable changes in the physical and chemical fractions of SOC were detected. These findings have greatly contributed to narrowing the knowledge gap regarding the understanding of the feedback between carbon cycle and climate change in these ecosystems. Nevertheless, there remain certain research insufficiencies.

**Figure 5.**
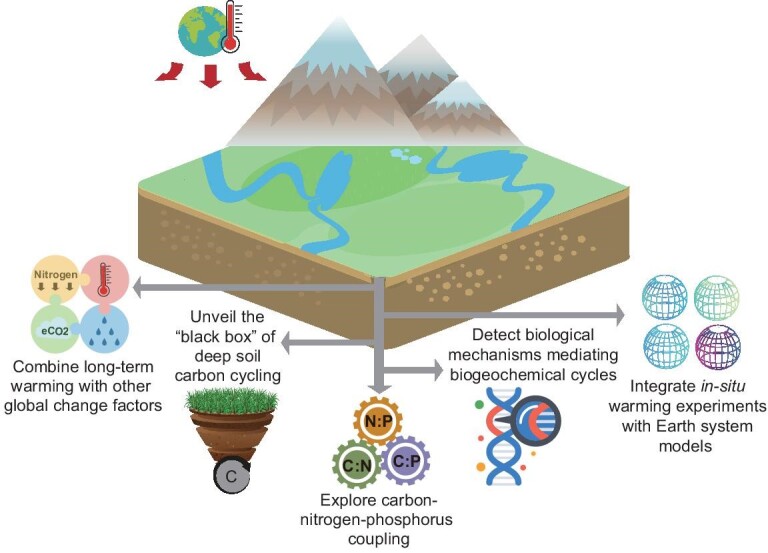
A conceptual diagram illustrating the future prospects of *in-situ* warming experiments across Tibetan alpine grasslands. Future studies can focus on deep soil carbon cycling and carbon-nitrogen-phosphorus coupling under warming scenarios, as well as investigate the biological mechanisms behind the responses of biogeochemical cycles to warming. Furthermore, integrating long-term warming experiments with Earth system models is highly necessary. The plant, soil profile, and double helix images were derived from 58pic.com.

Continuous long-term monitoring is essential to fully capture the effects of warming on the ecosystem carbon cycle in alpine grasslands, particularly when integrating other global change factors to simulate more realistic scenarios of environmental change (Fig. 5). Moreover, there remains a critical need to detect the response of deep soil carbon cycling to warming, and to explore the mechanisms by which nitrogen and phosphorus cycles regulate ecosystem carbon dynamics from a biological perspective (Fig. 5). Finally, *in-situ* warming experiments are expected to be coupled with Earth system models (ESMs) for improving the accuracy of predictions regarding future carbon-climate feedbacks in Tibetan alpine grasslands (Fig. 5).

### Combination of long-term warming with other global change factors

Long-term *in-situ* warming experiments are essential since ecosystem components’ responses to warming vary substantially with the duration of warming. Responses of vegetation growth [39], soil microbial physiological traits [168,169], and carbon fluxes [170–172] are highly time-dependent, and short-term studies risk providing a biased view of the impacts of climate warming on ecosystem carbon cycling [173]. However, in Tibetan alpine grasslands, only a few *in-situ* warming experiments have continued observations for more than 10 years [42,54,111,123]. Such lack of long-term data prevents the discovery of the potential thresholds or tipping points that may emerge during the long-term response to warming. Given that most of the existing warming experiments were initiated over a decade ago (42 out of 58; Table S1), we strongly recommend continued monitoring and reporting the long-term response of ecosystem carbon cycle to warming. In particular, newly established platforms using next-generation warming techniques (e.g. whole soil-profile and whole ecosystem warming) should prioritize long-term observational plans [25]. Furthermore, as discussed in previous Sections and supported by recent meta-analyses, the coupling of multiple global change factors (e.g. elevated CO_2_ concentration and altered precipitation regime) may have more pronounced effects on ecosystem carbon cycling than warming alone [174,175]. Therefore, building upon existing long-term warming experiments, it would be more advanced and representative to integrate one or more global change factors for a more realistic and comprehensive understanding of ecosystem responses to ongoing climate change across Tibetan alpine grasslands [176].

### Unveiling the ‘black box’ of carbon cycling processes in deep soils

Deep soils, alike surface soils, are experiencing progressive warming (Fig. 1b–e). This warming is especially pronounced in permafrost-distributed areas, where deep soil warming is usually accompanied by phase changes in the soil (e.g. permafrost thawing [25,177]). Particularly, over 70% of SOC is stored in deep soils (>30 cm) across Tibetan alpine grasslands, making changes in this stock crucial for modulating regional carbon-climate feedback [178]. However, traditional field warming techniques, which mostly employ a top-down approach, make warming deep soils particularly challenging [22,159]. As a result, the majority of studies on ecosystem carbon cycling in response to warming have primarily focused on the vegetation layer and topsoil, leaving deep soils comparatively underexplored (but see [13,123,133,149,179]). Encouragingly, the two newly established next-generation warming experiments in these alpine grasslands (i.e. whole soil-profile [24] and whole ecosystem warming [25]) have achieved a technological breakthrough in simulating deep soil warming and permafrost thawing. With these advancements, we strongly advocate for increased research on how deep-soil biogeochemical cycling processes respond to elevated temperature. Such efforts will inevitably generate critical new insights into deciphering the ‘black box’ processes occurring in the deep soils across Tibetan alpine grasslands.

### Carbon-nitrogen-phosphorus coupling under a warming scenario

Soil nutrients, notably nitrogen and phosphorus, are vital drivers of community structure and carbon-related functions [4,36]. However, in Tibetan alpine grasslands, studies have focused on a narrow spectrum of soil nitrogen transformation processes, such as net ammonification and net nitrification [41]. A systematic and holistic understanding of the *in-situ* response of soil nitrogen transformations to climatic warming remains sorely lacking. Several important issues, such as whether nitrogen cycling of alpine grasslands will become more open or closed in a warmer climate, and how these changes would impact ecosystem carbon cycling, remain inadequately understood [46]. Additionally, current studies concerning soil phosphorus cycling often rely on indirect methods (e.g. changes in phosphorus stock) [47,49]. Encouragingly, the recent advent of ^33^P-labelling techniques means that there is now a direct and quantitative tool available to explore the effects of warming on soil phosphorus transformations [180]. This new approach holds great potential for achieving groundbreaking insight into the relationships between soil phosphorus dynamics and the growth, reproduction, and carbon-related functioning of organisms in alpine grasslands. Taken together, given our limited knowledge of soil nitrogen and phosphorus dynamics and their potential roles in moderating ecosystem carbon cycle in these alpine grasslands, there is an urgent need for further endeavors regarding the coupling of carbon-nitrogen-phosphorus cycles in response to warming.

### Biological mechanisms mediating the response of biogeochemical cycle to climate warming

As *per* the aforementioned summary, experimental warming has been shown to substantially affect community structure and biogeochemical cycling in Tibetan alpine grasslands, primarily through inducing changes in environmental factors such as temperature and soil water status. To progress further, studies should now delve into the biological mechanisms mediating these responses to climate warming. For instance, the understanding of plant functional traits in response to climate change should extend beyond phenology, morphology, chemistry, and photosynthesis. Leaf metabolic traits, encompassing metabolites involved in cellular functions and produced to confront stress, not only provide biochemical validations for existing functional traits, but also complement the missing dimensions of plant life-history variation [181]. However, for Tibetan alpine grasslands, there is currently little understanding of how such metabolic traits respond to warming or how these changes modulate biogeochemical cycles. Similarly, only a small number of studies have utilized DNA-level metagenomic analyses to predict soil microbial functions [109,110]. A combination of such work with RNA-level approaches (e.g. metatranscriptome) would offer deeper insights into the mechanisms by which soil microbes adapt to climate change and moderate carbon cycling [182]. Additionally, it is also imperative to explore the responses of key bioindicators, such as microbial carbon use efficiency and thermal adaptation of soil microbial respiration, to warming. These bioindicators are pivotal for understanding SOC dynamics, including its formation, preservation, and loss within terrestrial ecosystems [183,184]. Overall, a comprehensive grasp of these biological mechanisms is expected to offer critical insights into how alpine grasslands respond to climate warming, helping to understand ecosystem carbon dynamics under warming scenarios.

### Integrating *in-situ* warming experiments with Earth system models

ESMs serve as essential tools for understanding long-term ecosystem dynamics in response to global change, as well as for assessing carbon-climate feedback at regional scales [3,185]. Despite their unique advantages, significant uncertainties persists in model simulations regarding the effects of past and future climate change on carbon cycling in Tibetan alpine grasslands [177]. These uncertainties may stem from the incomplete integration of key processes and mechanisms that respond to warming in these regions. To address this issue, we propose several strategies for integrating current data of *in-situ* warming experiments with models to provide more accurate predictions on the fate of ecosystem carbon cycling. On the one hand, understanding the mechanisms underlying ecosystem carbon cycle responses to warming through manipulative experiments can help to improve model parameterization and develop response functions that were previously treated as constants, thereby rendering models more rational [186]. In particular, uncovering the direct and indirect processes by which warming influences biogeochemical cycling (Figs. 2–4), and incorporating these processes into ESMs, can help to optimize model structure. These refinements are expected to be conducted in tandem with complementary studies within *in-situ* experiments, including laboratory incubations, isotope tracers, and genomics approaches [186,187]. On the other hand, measured datasets from *in-situ* treated plots can provide valuable validation data for model outputs, such as evapotranspiration, microbial biomass carbon, ecosystem carbon flux, and soil carbon stock. Using these datasets can further refine model parameters, ultimately improving the accuracy of model-simulated output. By reducing uncertainties in ESMs predictions through these efforts, we can achieve more reliable forecasts of ecosystem carbon cycle feedbacks to climate warming in Tibetan alpine grasslands [3].

## Supplementary Material

nwae371_Supplemental_File
