# Supplementary material for: Heating up the roof of the world: tracing the impacts of in-situ warming on carbon cycle in alpine grasslands on the Tibetan Plateau
Source: Natl Sci Rev. 2024 Nov 26;12(2):nwae371. doi: 10.1093/nsr/nwae371 (PMC11771398; doi:10.1093/nsr/nwae371)
Supplement: nwae371_Supplemental_File [file nwae371_supplemental_file.docx]

**Supporting Information for**

**Heating up the roof of the world: tracing the impacts of *in-situ* warming on carbon cycle in Tibetan alpine grasslands**

Yuxuan Bai^1,2^, Yunfeng Peng^1,2^, Dianye Zhang^1,2^, Guibiao Yang^1,2^, Leiyi Chen^1,2^, Luyao Kang^1,2,3^, Wei Zhou^1,2,3^, Bin Wei^1,2,3^, Yuhong Xie^1,2,3^, and Yuanhe Yang^1,2,3^

*^1^State Key Laboratory of Vegetation and Environmental Change, Institute of Botany, Chinese Academy of Sciences, Beijing 100093, China*

*^2^China National Botanical Garden, Beijing 100093, China.*

*^3^University of Chinese Academy of Sciences, Beijing 100049, China*

^*^**Corresponding author:** Dr. Yuanhe Yang, tel.: +86 10-6283 6638, E-mail: [yhyang@ibcas.ac.cn](mailto:yhyang@ibcas.ac.cn)

**Manuscript type:** Review

**Table S1** Information about the *in situ* warming experiments in alpine grasslands on the Tibetan Plateau.

| Site | Latitude | Longitude | Elevation (m) | MAT (℃) | MAP (mm) | Ecosystem | Method | Started year | Plot area (m^2^) | Height (m) | Reference |
| --- | --- | --- | --- | --- | --- | --- | --- | --- | --- | --- | --- |
| Haibei | 37°37' N | 101°12' E | 3200 | -1.6 | 500 | Alpine meadow | OTC | 1997 | 1.8 | 0.4 | [1] |
| Maqin | 34°17' N | 100°43' E | 4120 | -2.6 | 513 | Alpine meadow | OTC | 2002 | 0.9~2.1 | 0.4 | [2] |
| Fenghuo mountain | 34°44' N | 92°54' E | 4750 | -5.3 | 270 | Alpine meadow; Swamp meadow | OTC | 2006 | 1.0~2.0 | 0.4 | [3] |
| Beiluhe | 34°51' N | 92°57' E | 4659 | -3.6 | 424 | Swamp meadow | OTC | 2007 | 1.0~2.0 | 0.4~0.8 | [4] |
| Hongyuan | 32°48' N | 102°33' E | 3500 | 0.9 | 730 | Alpine meadow | OTC | 2007 | 4.0 | 2.0 | [5] |
| Beiluhe | 34°51' N | 92°57' E | 4659 | -3.8 | 383 | Alpine meadow; Swamp meadow | OTC | 2008 | 1.5 | 0.4 | [6] |
| Damxung | 30°30' N | 91°03' E | 4313 | 1.3 | 476.8 | Alpine meadow | OTC | 2008 | 2.0 | 0.4 | [7] |
| Hongyuan | 32°48' N | 102°33' E | 3500 | 1.1 | 690 | Alpine meadow | OTC | 2009 | 4.0 | 2.0 | [8] |
| Hongyuan | 32°48' N | 102°33' E | 3500 | 0.9 | 730 | Alpine meadow | OTC | 2009 | 1.0 | 1.0 | [9] |
| Nam Tso lake | 30°43' N | 91°03' E | 4875 | -0.7 | 407 | Alpine meadow | OTC | 2009 | 2.0 | 0.4 | [10] |
| Songpan | 32°51' N | 103°33' E | 3400 | 2.8 | 718 | Alpine meadow | OTC | 2009 | 1.7 | 1.5 | [11] |
| Baingoin | 31°23' N | 90°02' E | 4500 | -3 | 450 | Alpine steppe | OTC | 2010 | 2.0 | 0.4 | [12] |
| Damxung | 30°51' N | 91°05' E | 4333 | 1.3 | 477 | Alpine meadow | OTC | 2010 | 1.5 | 0.4 | [13] |
| Damxung | 30°31' N | 91°03' E | 4313~4693 | 1.3 | 476.8 | Alpine meadow | OTC | 2010 | 2.0 | 0.4 | [14] |
| Haibei | 36°57' N | 100°51' E | 3140 | 0.8 | 409 | Alpine meadow | OTC | 2010 | 2.0 | 0.4 | [15] |
| Xainza | 30°57' N | 88°42' E | 4675 | 0 | 300 | Alpine steppe | OTC | 2010 | 2.6 | 1.6 | [16] |
| Naqu | 31°26' N | 101°10' E | 4500 | -3 | 450 | Alpine meadow | OTC | 2010 | 2.0 | 0.4 | [17] |
| Baingoin | 31°23' N | 90°02' E | 4725 | -0.4 | 334 | Alpine steppe | OTC | 2011 | 1.1 | 0.5 | [18] |
| Haibei | 37°37' N | 101°17' E | 3220 | -1.6 | 560 | Alpine meadow | OTC | 2011 | 1.2~2.1 | 0.4 | [19] |
| Haibei | 37°36' N | 101°19' E | 3220 | -1.7 | 561 | Alpine meadow | OTC | 2011 | 2.1 | 1.5 | [20] |
| Hongyuan | 32°51' N | 103°33' E | 3500 | 2.8 | 718 | Alpine meadow | OTC | 2011 | 3.1 | 0.8 | [21] |
| Naqu | 31°26' N | 92°01' E | 4460 | -0.6 | 458 | Alpine meadow | OTC | 2011 | 1.1 | 0.5 | [18] |
| Naqu | 31°16' N | 92°06' E | 4453 | -1.2 | 430 | Alpine meadow | OTC | 2012 | 1.8 | 0.5 | [22] |
| Damxung | 30°30' N | 91°04' E | 4313 | 1.8 | 476 | Alpine meadow | OTC | 2013 | 2.0 | 0.4 | [23] |
| Damxung | 30°30' N | 91°03' E | 4313 | 1.3 | 477 | Alpine meadow | OTC | 2013 | 2.0 | 0.4 | [24] |
| Gangca | 37°45' N | 100°05' E | 3800 | -3.4 | 466 | Swamp meadow | OTC | 2013 | 2.1 | 0.5 | [25] |
| Gangca | 37°18' N | 100°15' E | 3280 | 0.1 | 387 | Alpine steppe | OTC | 2013 | 2.1 | 0.5 | [26] |
| Heihe | 38°48' N | 99°00' E | 3600~3800 | 1.0 | 1080 | Alpine meadow; Swamp meadow | OTC | 2013 | 1.0 | 0.3 | [27] |
| Naqu | 31°38' N | 92°1' E | 4600 | -1.2 | 430 | Alpine meadow | OTC | 2013 | 2.6~4.4 | 0.4~1.0 | [28] |
| Sanjiangyuan | 33°24' N | 97°18' E | 4270 | 0.9 | 562.2 | Alpine meadow | OTC | 2013 | 4.0 | 0.4 | [29] |
| Damxung | 30°30' N | 91°03' E | 4313 | 1.3 | 476.8 | Alpine meadow | OTC | 2014 | 2.0 | 0.4~0.8 | [30] |
| Gangca | 37°44' N | 100°05' E | 3800 | -3.0 | 420 | Swamp meadow | OTC | 2014 | 2.1 | 0.5 | [31] |
| Haibei | 36°55' N | 100°57' E | 3029 | 1.4 | 410 | Alpine meadow | OTC | 2014 | 2.2 | 0.7 | [32] |
| Hongyuan | 32°48' N | 102°33' E | 3500 | 0.9 | 730 | Alpine meadow | OTC | 2014 | 165.0 | 2.5 | [33] |
| Naqu | 31°38' N | 92°1' E | 4600 | -1.2 | 430 | Alpine meadow | OTC | 2014 | 10.9 | 2.0 | [34] |
| Aerjin mountain | 37°00' N | 89°00' E | 3100 | 1.4 | 350 | Alpine meadow | OTC | 2015 | 2.0 | 0.4 | [35] |
| Damxung | 30°30' N | 91°04' E | 4313 | 1.8 | 476 | Alpine meadow | OTC | 2015 | 2.0 | 0.4~0.8 | [36] |
| Gongga mountain | 29°52' N | 102°30' E | 3800~4300 | 4.0 | 1200 | Alpine meadow | OTC | 2015 | 1.0 | 0.4 | [37] |
| Gonghe | 37°02' N | 99°35' E | 3270 | 0 | 377 | Alpine steppe | OTC | 2015 | 2.0 | 0.4 | [38] |
| Haiyan | 36°56' N | 100°57' E | 3100 | 1.4 | 350 | Alpine meadow | OTC | 2015 | 2.0 | 0.4 | [38] |
| Maqu | 33°59' N | 102°00' E | 3538 | 1.2 | 620 | Alpine meadow | OTC | 2015 | 2.0 | 0.5 | [39] |
| Sygera Mountain | 29°40' N | 94°24' E | 4450 | 0.5 | 1134 | Alpine meadow | OTC | 2015 | 4.1 | 1.0 | [40] |
| Gongga mountain | 29°52' N | 102°30' E | 3800~4200 | 4.1 | 1691 | Alpine meadow | OTC | 2016 | NR | NR | [41] |
| Maqu | 33°40' N | 101°52' E | 3540 | 1.2 | 620~780 | Alpine meadow | OTC | 2017 | 0.7 | 0.5 | [42] |
| Maqu | 33°40' N | 101°52' E | 3500 | 2.2 | 450~780 | Alpine meadow | OTC | 2017 | 1.5 | 0.5 | [43] |
| Naqu | 31°49' N | 91°44' E | 4800 | 1.3 | 550 | Swamp meadow | OTC | 2018 | 4.4 | 0.4-0.8 | [44] |
| Hongyuan | 32°48' N | 102°33' E | 3500 | 0.9 | 730 | Alpine meadow | Greenhouse | 2013 | 150.0 | NR | [45] |
| Haibei | 37°37' N | 101°12' E | 3200 | -2.0 | 500 | Alpine meadow | Infrared heater | 2006 | 9.0 | 1.5 | [46] |
| Beiluhe | 34°49' N | 92°56' E | 4635 | -3.8 | 291 | Alpine meadow; Swamp meadow | Infrared heater | 2010 | 4.0 | 1.5 | [47] |
| Hongyuan | 32°84' N | 102°58' E | 3500 | 1.1 | 753 | Alpine meadow | Infrared heater | 2010 | 6.0 | 1.5 | [48] |
| Haibei | 37°37' N | 101°12' E | 3215 | -1.1 | 446 | Alpine meadow | Infrared heater | 2011 | 4.0 | 1.5 | [49] |
| Hongyuan | 32°48' N | 102°33' E | 3500 | 0.9 | 730 | Alpine meadow | Infrared heater | 2013 | 1.2 | 1.3 | [50] |
| Nam Co | 30°46' N | 90°59' E | 4730 | -0.6 | 414.6 | Alpine steppe | Infrared heater | 2013 | 2.9 | 1.3 | [51] |
| Hongyuan | 32°48' N | 102°33' E | 3500 | 0.9 | 730 | Alpine meadow | Infrared heater | 2015 | 4.0 | 1.2 | [52] |
| Haibei | 37°36' N | 101°119' E | 3215 | -1.1 | 488 | Alpine meadow | Infrared heater | 2019 | NR | 1.2 | [53] |
| Naqu | 31°49' N | 91°44' E | 4800 | 1.3 | 550 | Swamp meadow | Infrared heater | 2021 | 4.4 | 1.6 | [44] |
| Haibei | 37°37' N | 101°12' E | 3215 | -1.1 | 446 | Alpine meadow | Whole soil-profile | 2018 | 9.6 | -1~0 | [54] |
| Gangca | 37°30' N | 100°14' E | 3670 | -0.1 | 364.6 | Swamp meadow | Whole ecosystem | 2022 | 7.1 | -0.6~0 | [55] |

Note: MAT, mean annual temperature; MAP, mean annual precipitation; OTC, open top chamber; NR, not referred. The table is organized in the following orders: first, warming method; second, started year; third, site initials.

**REFERENCES**

1. Klein J, Harte J, Zhao X. Experimental warming causes large and rapid species loss, dampened by simulated grazing, on the Tibetan Plateau. *Ecology Letters*. 2004; **7**: 1170-1179. doi: 10.1111/j.1461-0248.2004.00677.x
2. Wang C, Wang G, Wang Y *et al.* Effects of long-term experimental warming on plant community properties and soil microbial community composition in an alpine meadow. *Israel Journal of Ecology & Evolution*. 2017; **63**: 85-96.
3. Li N, Wang G, Yang Y *et al.* Plant production, and carbon and nitrogen source pools, are strongly intensified by experimental warming in alpine ecosystems in the Qinghai-Tibet Plateau. *Soil Biology and Biochemistry*. 2011; **43**: 942-953. doi: 10.1016/j.soilbio.2011.01.009
4. Wang J, Wu Q. Impact of experimental warming on soil temperature and moisture of the shallow active layer of wet meadows on the Qinghai-Tibet Plateau. *Cold Regions Science and Technology*. 2013; **90-91**: 1-8. doi: 10.1016/j.coldregions.2013.03.005
5. Li G, Liu Y, Frelich L *et al.* Experimental warming induces degradation of a Tibetan alpine meadow through trophic interactions. *Journal of Applied Ecology*. 2011; **48**: 659-667. doi: 10.1111/j.1365-2664.2011.01965.x
6. Zhang B, Chen S, He X *et al.* Responses of soil microbial communities to experimental warming in alpine grasslands on the Qinghai-Tibet Plateau. *PLOS ONE*. 2014; **9**: e103859. doi: 10.1371/journal.pone.0103859
7. Fu G, Shen Z, Zhang X *et al.* Response of ecosystem respiration to experimental warming and clipping at daily time scale in an alpine meadow of tibet. *Journal of Mountain Science*. 2013; **10**: 455-463. doi: 10.1007/s11629-013-2360-y
8. Zi H, Hu L, Wang C *et al.* Responses of soil bacterial community and enzyme activity to experimental warming of an alpine meadow. *European Journal of Soil Science*. 2018; **69**: 429-438. doi: 10.1111/ejss.12547
9. Wu X, Duffy J, Reich P *et al.* A brown-world cascade in the dung decomposer food web of an alpine meadow: effects of predator interactions and warming. *Ecological Monographs*. 2011; **81**: 313-328. doi: 10.1890/10-0808.1
10. Dorji T, Totland Ø, Moe S *et al.* Plant functional traits mediate reproductive phenology and success in response to experimental warming and snow addition in Tibet. *Global Change Biology*. 2013; **19**: 459-472. doi: 10.1111/gcb.12059
11. Shi F, Chen H, Chen H *et al.* The combined effects of warming and drying suppress CO_2_ and N_2_O emission rates in an alpine meadow of the eastern Tibetan Plateau. *Ecological Research*. 2012; **27**: 725-733. doi: 10.1007/s11284-012-0950-8
12. Zhang Y, Gao Q, Dong S *et al.* Effects of grazing and climate warming on plant diversity, productivity and living state in the alpine rangelands and cultivated grasslands of the Qinghai-Tibetan Plateau. *The Rangeland Journal*. 2015; **37**: 57-65. doi: 10.1071/RJ14080
13. Zong N, Chai X, Shi P *et al.* Effects of warming and nitrogen addition on plant photosynthate partitioning in an alpine meadow on the Tibetan Plateau. *Journal of Plant Growth Regulation*. 2018; **37**: 803-812. doi: 10.1007/s00344-017-9775-6
14. Fu G, Shen Z, Zhang X *et al.* Response of soil microbial biomass to short-term experimental warming in alpine meadow on the Tibetan Plateau. *Applied Soil Ecology*. 2012; **61**: 158-160. doi:10.1016/j.apsoil.2012.05.002
15. Chen J, Luo Y, Xia J *et al.* Differential responses of ecosystem respiration components to experimental warming in a meadow grassland on the Tibetan Plateau. *Agricultural and Forest Meteorology*. 2016; **220**: 21-29. doi: 10.1016/j.agrformet.2016.01.010
16. Lu X, Fan J, Yan Y *et al.* Responses of soil CO_2_ fluxes to short-term experimental warming in alpine steppe ecosystem, northern Tibet. *PLOS ONE*. 2013; **8**: e59054. doi: 10.1371/journal.pone.0059054
17. Wang X, Dong S, Gao Q *et al.* Effects of short-term and long-term warming on soil nutrients, microbial biomass and enzyme activities in an alpine meadow on the Qinghai-Tibet Plateau of China. *Soil Biology and Biochemistry*. 2014; **76**: 140-142. doi: 10.1016/j.soilbio.2014.05.014
18. Ganjurjav H, Gao Q, Gornish E *et al.* Differential response of alpine steppe and alpine meadow to climate warming in the central Qinghai–Tibetan Plateau. *Agricultural and Forest Meteorology*. 2016; **223**: 233-240. doi: 10.1016/j.agrformet.2016.03.017
19. Shi G, Yao B, Liu Y *et al.* The phylogenetic structure of AMF communities shifts in response to gradient warming with and without winter grazing on the Qinghai–Tibet Plateau. *Applied Soil Ecology*. 2017; **121**: 31-40. doi: 10.1016/j.apsoil.2017.09.010
20. Wu Y, Zhou H, Chen W *et al.* Response of the soil food web to warming and litter removal in the Tibetan Plateau, China. *Geoderma*. 2021; **401**: 115318. doi: 10.1016/j.geoderma.2021.115318
21. Shi C, Sun G, Zhang H *et al.* Effects of warming on chlorophyll degradation and carbohydrate accumulation of alpine herbaceous species during plant senescence on the Tibetan Plateau. *PLOS ONE*. 2014; **9**: e107874. doi: 10.1371/journal.pone.0107874
22. Cui S, Meng F, Suonan J *et al.* Responses of phenology and seed production of annual Koenigia islandica to warming in a desertified alpine meadow. *Agricultural and Forest Meteorology*. 2017; **247**: 376-384. doi: 10.1016/j.agrformet.2017.08.034
23. Wang J, Fu G, Zhang G *et al.* The effect of higher warming on vegetation indices and biomass production is dampened by greater drying in an alpine meadow on the Northern Tibetan Plateau. *Journal of Resources and Ecology*. 2017; **8**: 105-112. doi: 10.5814/j.issn.1674-764x.2017.01.013
24. Shen Z, Li Y, Fu G. Response of soil respiration to short-term experimental warming and precipitation pulses over the growing season in an alpine meadow on the Northern Tibet. *Applied Soil Ecology*. 2015; **90**: 35-40. doi: 10.1016/j.apsoil.2015.01.015
25. Li F, Peng Y, Natali S *et al.* Warming effects on permafrost ecosystem carbon fluxes associated with plant nutrients. *Ecology*. 2017; **98**: 2851-2859. doi: 10.1002/ecy.1975
26. Li F, Peng Y, Zhang D *et al.* Leaf area rather than photosynthetic rate determines the response of ecosystem productivity to experimental warming in an alpine steppe. *Journal of Geophysical Research: Biogeosciences*. 2019; **124**: 2277-2287. doi: 10.1029/2019JG005193
27. Mu C, Zhang T, Zhao Q *et al.* Permafrost affects carbon exchange and its response to experimental warming on the northern Qinghai-Tibetan Plateau. *Agricultural and Forest Meteorology*. 2017; **247**: 252-259. doi: 10.1016/j.agrformet.2017.08.009
28. Zhu J, Zhang Y, Wang W. Interactions between warming and soil moisture increase overlap in reproductive phenology among species in an alpine meadow. *Biology Letters*. 2016; **12**: 20150749. doi: 10.1098/rsbl.2015.0749
29. Wang Z, Feng K, Lu G *et al.* Homogeneous selection and dispersal limitation dominate the effect of soil strata under warming condition. *Frontiers in Microbiology*. 2022; **13**: 801083. doi: 10.3389/fmicb.2022.801083
30. Fu G, Shen Z, Zhang X. Increased precipitation has stronger effects on plant production of an alpine meadow than does experimental warming in the Northern Tibetan Plateau. *Agricultural and Forest Meteorology*. 2018; **249**: 11-21. doi: 10.1016/j.agrformet.2017.11.017
31. Yuan X, Chen Y, Qin W *et al.* Plant and microbial regulations of soil carbon dynamics under warming in two alpine swamp meadow ecosystems on the Tibetan Plateau. *Science of The Total Environment*. 2021; **790**: 148072. doi: 10.1016/j.scitotenv.2021.148072
32. Wei X, Shi Y, Qin F *et al.* Effects of experimental warming, precipitation increase and their interaction on AM fungal community in an alpine grassland of the Qinghai-Tibetan Plateau. *European Journal of Soil Biology*. 2021; **102**: 103272. doi: 10.1016/j.ejsobi.2020.103272
33. Hu X, Zhou W, Sun S. Responses of plant reproductive phenology to winter-biased warming in an alpine meadow. *Frontiers in Plant Science*. 2020; **11**: 534703. doi: 10.3389/fpls.2020.534703
34. Zhu J, Zhang Y, Jiang L. Experimental warming drives a seasonal shift of ecosystem carbon exchange in Tibetan alpine meadow. *Agricultural and Forest Meteorology*. 2017; **233**: 242-249. doi: 10.1016/j.agrformet.2016.12.005
35. Kwaku E, Dong S, Shen H *et al.* Biomass and species diversity of different alpine plant communities respond differently to nitrogen deposition and experimental warming. *Plants* 2021; **10**: 2719. doi: 10.3390/plants10122719
36. Fu G, Zhang H, Sun W. Response of plant production to growing/non-growing season asymmetric warming in an alpine meadow of the Northern Tibetan Plateau. *Science of The Total Environment*. 2019; **650**: 2666-2673. doi: 10.1016/j.scitotenv.2018.09.384
37. Sun F, Chang R, Tariq A *et al.* Livestock grazing-exclusion under global warming scenario decreases phosphorus mineralization by changing soil food web structure in a Tibetan alpine meadow. *Science of The Total Environment*. 2023; **873**: 162313. doi: 10.1016/j.scitotenv.2023.162313
38. Zhao Z, Dong S, Jiang X *et al.* Effects of warming and nitrogen deposition on CH_4_, CO_2_ and N_2_O emissions in alpine grassland ecosystems of the Qinghai-Tibetan Plateau. *Science of The Total Environment*. 2017; **592**: 565-572. doi: 10.1016/j.scitotenv.2017.03.082
39. Shu M, Zhao Q, Li Z *et al.* Effects of global change factors and living roots on root litter decomposition in a Qinghai-Tibet alpine meadow. *Scientific Reports*. 2019; **9**: 16924. doi: 10.1038/s41598-019-53450-5
40. Wang G, Baskin C, Baskin J *et al.* Effects of climate warming and prolonged snow cover on phenology of the early life history stages of four alpine herbs on the southeastern Tibetan Plateau. *American Journal of Botany*. 2018; **105**: 967-976. doi: 10.1002/ajb2.1104
41. Yu J, Bing H, Chang R *et al.* Microbial metabolic limitation response to experimental warming along an altitudinal gradient in alpine grasslands, eastern Tibetan Plateau. *Catena*. 2022; **214**: 106243. doi: 10.1016/j.catena.2022.106243
42. Chen Y, Collins S, Zhao Y *et al.* Warming reduced flowering synchrony and extended community flowering season in an alpine meadow on the Tibetan Plateau. *Ecology*. 2023; **104**: e3862. doi: 10.1002/ecy.3862
43. Wang J, Bonser S, Liu K *et al.* Warming affects herbaceous germination, early survival, and growth by shifting plant-soil microbe interactions in an alpine ecosystem. *Plant and Soil*. 2023; **487**: 249-265. doi: 10.1007/s11104-023-05921-y
44. Yan Z, Wang T, Ding J *et al.* No slowdown of growing season extension with warming in a permafrost-affected meadow on the Tibetan Plateau. *Journal of Ecology*. 2024; **112**: 1774-1786. doi: 10.1111/1365-2745.14359
45. Zhao J, Yang Y, Xi X *et al.* Artificial warming facilitates growth but not survival of plateau frog (*Rana kukunoris*) tadpoles in presence of gape-limited predatory beetles. *PLOS ONE*. 2014; **9**: e98252. doi: 10.1371/journal.pone.0098252
46. Luo C, Xu G, Wang Y *et al.* Effects of grazing and experimental warming on DOC concentrations in the soil solution on the Qinghai-Tibet plateau. *Soil Biology and Biochemistry*. 2009; **41**: 2493-2500. doi: 10.1016/j.soilbio.2009.09.006
47. Peng F, Xue X, You Q *et al.* Warming effects on carbon release in a permafrost area of Qinghai-Tibet Plateau. *Environmental Earth Sciences*. 2015; **73**: 57-66. doi: 10.1007/s12665-014-3394-3
48. Quan Q, Zhang F, Tian D *et al.* Transpiration Dominates Ecosystem Water-Use Efficiency in Response to Warming in an Alpine Meadow. *Journal of Geophysical Research: Biogeosciences*. 2018; **123**: 453-462. doi: 10.1002/2017JG004362
49. Lin L, Zhu B, Chen C *et al.* Precipitation overrides warming in mediating soil nitrogen pools in an alpine grassland ecosystem on the Tibetan Plateau. *Scientific Reports*. 2016; **6**: 31438. doi: 10.1038/srep31438
50. Xi X, Li D, Peng Y *et al.* Experimental warming and precipitation interactively modulate the mortality rate and timing of spring emergence of a gallmaking *Tephritid* fly. *Scientific Reports*. 2016; **6**: 32284. doi: 10.1038/srep32284
51. Zhao J, Tian L, Wei H *et al.* Negative responses of ecosystem autotrophic and heterotrophic respiration to experimental warming in a Tibetan semi-arid alpine steppe. *Catena*. 2019; **179**: 98-106. doi: 10.1016/j.catena.2019.04.002
52. Xi X, Wu X, Nylin S *et al.* Body size response to warming: time of the season matters in a tephritid fly. *Oikos*. 2016; **125**: 386-394. doi: 10.1111/oik.02521
53. Jiang S, Xiao B, Fan X *et al.* Roles of plants in controlling the response of soil bacterial community to climate warming on the Qinghai-Tibetan Plateau. *European Journal of Soil Biology*. 2022; **110**: 103401. doi: 10.1016/j.ejsobi.2022.103401
54. Zhu B, Chen Y. Techniques and methods for field warming manipulation experiments in terrestrial ecosystems. *Chinese Journal of Plant Ecology*. 2020; **44**: 330-339. doi: 10.17521/cjpe.2019.0179
55. Bai Y, Peng Y, Zhou W *et al.* SWAMP: A new experiment for simulating permafrost warming and active layer deepening on the Tibetan Plateau. *Methods in Ecology and Evolution*. 2023; **14**: 1732-1746. doi: 10.1111/2041-210X.14124
